# Supplementary material for: Spatial Variation in Stream-Water Bacterial Communities Across Sites Differing in Wild Chinese Giant Salamander Detection Frequency
Source: Biology (Basel). 2026 Sep 18;15(18):1647. doi: 10.3390/biology15181647 (PMC13604818; doi:10.3390/biology15181647)
Supplement: Supplementary file 1 [file biology-15-01647-s001.zip › Data Related to “Spatial Variation in Stream-Water Bacterial Communities across Sites Differing in Wild Chinese Giant Salamander Detection Frequency”/Microbial Data/report/src/hyperlinks/ReadMe_qiime2_SRS_CH.html]

| result |  |
| --- | --- |
| \|-- 00.CleanData【若00.CleanData为压缩包，解压后链接生效】 | 【质控后可用于后续分析的有效数据】 |
| \| \|-- data\_table.xls | 【数据预处理统计及质控信息表】 |
| \| \|-- Sample\_Name | 【每个样品的质控结果】 |
| \| \| \|-- \*.effective.fastq.gz | 【去除嵌合体之后的fastq序列】 |
| \| \| \|-- \*.fna.gz | 【去除嵌合体之后的fasta序列】 |
| \|-- 01.RawData【若01.RawData为压缩包，解压后链接生效】 | 【原始下机数据和原始拼接后数据】 |
| \| \|-- Sample\_Name | 【每个样品对应的原始下机数据和原始拼接后数据】 |
| \| \| \|-- \*.extendedFrags.fastq | 【原始下机的 reads 拼接后的序列】 |
| \| \| \|-- \*\_1.fastq.gz | 【reads1 去除 baraode 和 primer 后得到的序列】 |
| \| \| \|-- \*\_2.fastq.gz | 【reads2 去除 barcode 和 primer 后得到的序列】 |
| \| \| \|-- \*.raw\_1.fastq.gz | 【reads1 未去除 baraode 和 primer 的原始序列】 |
| \| \| \|-- \*.raw\_2.fastq.gz | 【reads2 未去除 baraode 和 primer 的原始序列】 |
| \| \|-- data\_table.xls | 【数据预处理统计及质控信息表】 |
| \|-- 02.FeatureAnalysis | 【特征序列和特征表】 |
| \| \|-- aligned-rep-seqs.qza | 【多序列比对结果】 |
| \| \|-- denoisingTable\_qza | 【去噪丰度qza导出文件】 |
| \| \|-- denoisingTable.qza | 【去噪丰度qza文件】 |
| \| \|-- denoisingTable\_qzv | 【去噪丰度qzv导出文件】 |
| \| \|-- denoisingTable.qzv | 【去噪丰度qzv文件】 |
| \| \|-- feature.fasta | 【代表序列】 |
| \| \|-- featureSeqs\_qza | 【代表序列qza导出文件】 |
| \| \|-- ffeatureSeqs.qza | 【代表序列qza文件】 |
| \| \|-- featureSeqs\_qzv | 【代表序列qzv导出文件】 |
| \| \|-- featureSeqs.qzv | 【代表序列qzv文件】 |
| \| \|-- featureTable\_qza | 【特征表丰度qza导出文件】 |
| \| \|-- featureTable.qza | 【特征表丰度qza文件】 |
| \| \|-- featureTable\_qzv | 【特征表丰度qzv导出文件】 |
| \| \|-- featureTable.qzv | 【特征表丰度qzv文件】 |
| \| \|-- feature.tax\_assignments.txt | 【物种注释结果】 |
| \| \|-- masked-aligned-rep-seqs.qza | 【过滤去除高变区后的多序列比对结果】 |
| \| \|-- rooted-tree\_qza | 【有根树qza导出文件】 |
| \| \|-- rooted-tree.qza | 【有根树qza文件】 |
| \| \|-- unrooted-tree\_qza | 【无根树qza导出文件】 |
| \| \|-- unrooted-tree.qza | 【无根树qza文件】 |
| \| \|-- seq\_taxonomy\_qza | 【物种注释qza导出文件】 |
| \| \|-- seq\_taxonomy.qza | 【物种注释qza文件】 |
| \| \|-- seq\_taxonomy\_qzv | 【物种注释qzv导出文件】 |
| \| \|-- seq\_taxonomy.qzv | 【物种注释qzv文件】 |
| \| \|-- tables | 【特征表】 |
| \| \| \|-- sample/group1/... | 【样本及分组的特征表】 |
| \| \| \| \|-- featureTable\_Absolute | 【各（样本,组）绝对丰度特征表】 |
| \| \| \| \| \|-- featureTable.\*.\*.absolute.txt | 【各（样本,组）总界门纲目科属种（total,k,p,c,o,f,g,s）水平上的绝对丰度】 |
| \| \| \| \|-- featureTable\_Relative | 【各（样本,组）相对丰度特征表】 |
| \| \| \| \| \|-- featureTable.\*.\*.relative.xls | 【各（样本,组）总界门纲目科属种（total,k,p,c,o,f,g,s）水平上的相对丰度】 |
| \| \| \| \|-- featureTable\_Even | 【各（样本,组）均一化处理后的绝对丰度特征表（均一化处理使每个样品的各个 OTU/ASV 丰度之和相等）】 |
| \| \| \| \| \|-- featureTable.\*.total.even.txt | 【各（样本,组）均一化处理后的绝对丰度】 |
| \| \| \| \| \|-- featureTable.\*.\*.absolute.xls | 【各（样本,组）界门纲目科属种（k,p,c,o,f,g,s）水平上均一化处理后的绝对丰度】 |
| \| \| \| \|-- featureTable\_Biom | 【各（样本,组）biom文件】 |
| \| \| \| \| \|-- featureTable.\*.total.absolute.txt.biom | 【各（样本,组）的绝对丰度 biom表】 |
| \| \| \| \| \|-- featureTable.\*.total.even.txt.biom | 【各（样本,组）均一化处理后的绝对丰度 biom表】 |
| \| \| \| \| \|-- featureTable.\*.total.relative.txt.biom | 【各（样本,组）的相对丰度 biom表】 |
| \| \| \| \|-- featureTable\_tableStat | 【各（样本,组）注释结果统计】 |
| \| \| \| \| \|-- featureTable.briefStatInfo.txt | 【各（样本,组）简版注释统计结果】 |
| \| \| \| \| \|-- featureTable.relativeStat.\*.xls | 【各（样本,组）各层级注释数量统计】 |
| \| \| \| \| \|-- classifiedStat.sample.absolute.{xls,svg,png} | 【各（样本,组）各层级注释统计结果及可视化展示】 |
| \| \| \| \| \|-- featureTable.\*.Tags\_stat.xls | 【各（样本,组）Tags统计结果】 |
| \| \| \| \| \|-- featureTable.\*.TagsDistribution.{svg,,png}} | 【各（样本,组）Tags统计结果可视化展示】 |
| \|-- 03.TaxonVisual | 【注释结果可视化】 |
| \| \|-- barplot | 【物种相对丰度柱形图展示】 |
| \| \| \|-- sample/group1/... | 【样本及分组的物种相对丰度柱形图】 |
| \| \| \| \|-- top10.\*.\*.barplot.{xls,png,svg} | 【各（样本,组）门纲目科属种（p,c,o,f,g,s）水平上的 top10 物种相对丰度柱形图】 |
| \| \| \| \|-- top30.\*.g.barplot.{xls,png,svg} | 【各（样本,组）属(g)水平的top30物种相对丰度柱形图】 |
| \| \|-- heatmap | 【物种注释聚类热图】 |
| \| \| \|-- sample/group1/... | 【样本及分组的物种注释聚类热图】 |
| \| \| \| \|-- heatmap.\*.\*.{pdf,png,txt} | 【各（样本,组）门纲目科属种（p,c,o,f,g,s）水平上的物种聚类热图】 |
| \| \|-- tree100 | 【属水平top100物种进化树圈图】 |
| \| \| \|-- sample/group1/... | 【样本及分组的属水平top100物种进化树圈图】 |
| \| \| \| \|-- genus\_100.tree | 【属水平top100物种进化树】 |
| \| \| \| \|-- genus\_100.tree.{png,svg} | 【属水平top100物种进化树圈图】 |
| \| \|-- venn | 【基于 OTU/ASV 的Venn图】 |
| \| \| \|-- sample/group1/... | 【样本及分组基于 OTU/ASV 的Venn图】 |
| \| \| \| \|-- \* | 【进行venn图分析的（样本,组）名字，以-连接，例A-B】 |
| \| \| \| \| \|-- \*\_\*.txt | 【（样本,组） 特有的OTU/ASV,例 A-B\_A.txt(A特有)】 |
| \| \| \| \| \|-- \*\_\*:\*.txt | 【（样本,组） 共有的OTU/ASV,例 A-B\_A：B.txt(A,B共有)】 |
| \| \| \| \| \|-- \*.{png,svg} | 【（样本,组）Venn图展示】 |
| \| \|-- flower | 【基于 OTU/ASV 的花瓣图】 |
| \| \| \|-- sample/group1/... | 【样本及分组基于 OTU/ASV 的花瓣图】 |
| \| \| \| \|-- \* | 【进行花瓣图分析的（样本,组）名字，以-连接，例A-B-C-D-E-F】 |
| \| \| \| \| \|-- \*\_all-core.xls | 【（样本,组） 共有的OTU/ASV,例 A-B-C-D-E-F\_all-core.xls(共有OTU/ASV)】 |
| \| \| \| \| \|-- \*\_\*.xls | 【每个（样本,组） 特有的OTU/ASV,例 A-B-C-D-E-F\_A.xls(A特有)】 |
| \| \| \| \| \|-- \*.{xls,png,svg} | 【（样本,组）花瓣图作图数据及可视化展示】 |
| \| \|-- ternary | 【三元相图分析结果】 |
| \| \| \|-- sample/group1/... | 【样本及分组三元相图分析结果】 |
| \| \| \| \|-- \*/\*.ternary.{jpeg,pdf} | 【各（样本,组）门纲目科属种（p,c,o,f,g,s）水平上的三元相图可视化展示】 |
| \|-- 04.AlphaDiversity | 【alpha 多样性分析结果】 |
| \| \|-- alpha\_index\_table | 【alpha 多样性指数】 |
| \| \| \|-- sample/group1/... | 【样本及组 alpha 多样性指数】 |
| \| \| \| \|-- alpha\_diversity\_index.{sample,group}.txt | 【 alpha 多样性指数】 |
| \| \|-- visual\_specaccum | 【物种累积曲线】 |
| \| \| \|-- sample | 【样本的物种累积曲线】 |
| \| \| \| \|-- specaccum\_test.{pdf,png} | 【（样本,组）物种累积曲线图】 |
| \| \|-- visual\_rankAbundance | 【Rank Abundance 曲线】 |
| \| \| \|-- sample/group1/... | 【样本及组 Rank Abundance 曲线】 |
| \| \| \| \|-- rank\_abundance.{sample,group}.{pdf,png} | 【 Rank Abundance 曲线展示】 |
| \| \|-- visual\_rarefaction | 【稀疏性曲线】 |
| \| \| \|-- sample/group1/... | 【样本及组 稀疏性曲线】 |
| \| \| \| \|-- \*/\*.{sample,group}.{pdf,png,xls} | 【(chao1,goods\_coverage,observed\_species,PD\_whole\_tree,shannon,simpson)稀疏性曲线展示】 |
| \| \|-- visual\_alphaDiversity | 【alpha 多样性组间比较箱型图】 |
| \| \| \|-- group1/... | 【组间比较箱型图】 |
| \| \| \| \|-- \* | 【组间 (ACE,chao1,goods\_coverage,observed\_species,PD\_whole\_tree,shannon,simpson) 比较箱型图】 |
| \| \| \| \| \|-- \*.{pdf,png} | 【指数箱型图】 |
| \| \| \| \| \|-- \*\_kruskalWallis.{txt,pdf,png} | 【多组间非参数 kruskalWallis 检验及可视化（组内样本>2,组>2）】 |
| \| \| \| \| \|-- \*\_Tukey.{txt,pdf,png} | 【多组间方差 Tukey 分析及可视化（组内样本>2,组>2）】 |
| \| \| \| \| \|-- \*\_test.{txt,pdf,png} | 【组间 T-test 检验及可视化（组内样本>2,组=2）】 |
| \| \| \| \| \|-- \*\_wilcox.{txt,pdf,png} | 【组间 wilcox秩和检验及可视化（组内样本>2,组=2）】 |
| \|-- 05.BetaDiversity | 【Beta 多样性分析结果】 |
| \| \|-- beta\_distance | 【Beta 距离分析结果】 |
| \| \| \|-- sample/group1/... | 【样本及组 Beta 距离分析结果】 |
| \| \| \| \|-- \*\_distance\_matrix\_qza | 【(bray\_curtis,jaccard,(un)weighted\_unifrac) 距离qza导出文件】 |
| \| \| \| \|-- \*\_distance\_matrix.qza | 【(bray\_curtis,jaccard,(un)weighted\_unifrac) 距离qza文件】 |
| \| \| \| \|-- \*\_pcoa\_results\_qza | 【(bray\_curtis,jaccard,(un)weighted\_unifrac) PCoA 分析qza导出文件】 |
| \| \| \| \|-- \*\_pcoa\_results.qza | 【(bray\_curtis,jaccard,(un)weighted\_unifrac) PCoA 分析qza文件】 |
| \| \|-- beta\_upgma | 【Beta UPGMA 分析结果】 |
| \| \| \|-- sample/group1/... | 【样本及组 Beta UPGMA 分析结果】 |
| \| \| \| \|-- (un)weighted\_unifrac | 【基于(非)加权 unifrac 距离 Beta UPGMA 分析结果】 |
| \| \| \| \| \|-- (un)weighted\_unifrac.{sample,group}\_upgma.tre | 【基于(非)加权 unifrac 距离的 UPGMA 聚类树文件，MEGA 软件可以打开】 |
| \| \| \| \| \|-- (un)weighted\_unifrac.{sample,group}.{pdf,png} | 【基于(非)加权 unifrac 距离的 UPGMA 聚类树图】 |
| \| \| \| \| \|-- upgam.(un)weighted\_unifrac.{sample,group}.tree.{svg,png} | 【基于(非)加权 unifrac 距离的 UPGMA 聚类树合并图】 |
| \| \|-- beta\_pca | 【Beta PCA 分析结果】 |
| \| \| \|-- sample/group1/... | 【样本及组 Beta PCA 分析结果】 |
| \| \| \| \|-- PCA.{xls,pdf,png} | 【 PCA 作图数据及未标样品名称的 PCA 图】 |
| \| \| \| \|-- PCA\_lable.{pdf,png} | 【标有样品名称的 PCA 图】 |
| \| \| \| \|-- PCA\_circle.{pdf,png} | 【未标样本名带有置信椭圆的 PCA 图】 |
| \| \| \| \|-- PCA\_circleLable.{pdf,png} | 【标有样本名带有置信椭圆的 PCA 图】 |
| \| \|-- beta\_pcoa | 【Beta PCoA 分析结果】 |
| \| \| \|-- sample/group1/... | 【样本及组 Beta PCoA 分析结果】 |
| \| \| \| \|-- PCoA\_display.{pdf,png} | 【 基于(非)加权 unifrac 距离的 PCoA 合并展示图】 |
| \| \| \| \|-- \* | 【 基于（(un)weighted\_unifrac,binary\_jaccard,bray\_curtis）PCoA 分析结果】 |
| \| \| \| \| \|-- PCoA.{xls,pdf,png} | 【 PCoA 作图数据及未标样品名称的 PCoA 图】 |
| \| \| \| \| \|-- PCoA\_label.{pdf,png} | 【标有样品名称的 PCoA 图】 |
| \| \| \| \| \|-- PCoA\_circle.{pdf,png} | 【未标样本名带有置信椭圆的 PCoA 图】 |
| \| \| \| \| \|-- PCoA\_circleLabel.{pdf,png} | 【标有样本名带有置信椭圆的 PCoA 图】 |
| \| \| \| \| \|-- emperor\_pcoa\_plots | 【三维 PCoA 网页版展示】 |
| \| \|-- beta\_nmds | 【Beta NMDS 分析结果】 |
| \| \| \|-- sample/group1/... | 【样本及组 Beta NMDS 分析结果】 |
| \| \| \| \|-- NMDS\_display.{pdf,png} | 【 基于(非)加权 unifrac 距离的 NMDS 合并展示图】 |
| \| \| \| \|-- \* | 【 基于（(un)weighted\_unifrac,binary\_jaccard,bray\_curtis）NMDS 分析结果】 |
| \| \| \| \| \|-- \*.NMDS.{xls,pdf,png} | 【 NMDS 作图数据及未标样品名称的 NMDS 图】 |
| \| \| \| \| \|-- \*.NMDS\_lable.{pdf,png} | 【标有样品名称的 NMDS 图】 |
| \| \| \| \| \|-- \*.NMDS\_circle.{pdf,png} | 【未标样本名带有置信椭圆的 NMDS 图】 |
| \| \| \| \| \|-- \*.NMDS\_circleLable.{pdf,png} | 【标有样本名带有置信椭圆的 NMDS 图】 |
| \| \|-- beta\_dca | 【Beta DCA 分析结果】 |
| \| \| \|-- sample/group1/... | 【样本及组 Beta DCA 分析结果】 |
| \| \| \| \|-- DCA.{xls,pdf,png} | 【 DCA 作图数据及 DCA 图】 |
| \| \|-- beat\_unifracHeatmap | 【Beta unifrac 距离热图】 |
| \| \| \|-- sample/group1/... | 【样本及组 Beta unifrac 距离热图】 |
| \| \| \| \|-- beta\_diversity.heatmap.{svg,png} | 【包含(un)Weighted 两种距离的热图】 |
| \| \| \| \|-- beta\_diversity.heatmap.(un)Weighted.{svg,png} | 【(非)加权 unifrac 距离热图】 |
| \| \|-- beta\_div | 【组间 Beta 多样性比较箱型图】 |
| \| \| \|-- group1/... | 【组间 Beta 多样性比较箱型图】 |
| \| \| \| \|-- (un)weighted\_unifrac | 【基于(非)加权 unifrac 距离的组间 Beta 多样性比较箱型图】 |
| \| \| \| \| \|-- (un)weighted\_unifrac.{pdf,png} | 【基于(非)加权 unifrac 距离的 Beta 多样性箱型图】 |
| \| \| \| \| \|-- (un)weighted\_unifrac\_kruskalWallis\_all.txt | 【多组间非参数 kruskalWallis 检验（组内样本>2,组>2）】 |
| \| \| \| \| \|-- (un)weighted\_unifrac\_kruskalWallis.{pdf,png} | 【多组间非参数 kruskalWallis 检验可视化展示（组内样本>2,组>2）】 |
| \| \| \| \| \|-- (un)weighted\_unifrac\_TukeyHSD\_all.txt | 【多组间方差 TukeyHSD 分析（组内样本>2,组>2）】 |
| \| \| \| \| \|-- (un)weighted\_unifrac\_TukeyHSD.{pdf,png} | 【多组间方差 TukeyHSD 分析可视化展示（组内样本>2,组>2）】 |
| \| \| \| \| \|-- (un)weighted\_unifrac\_tTest\_all.txt | 【组间 T-test 检验（组内样本>2,组=2）】 |
| \| \| \| \| \|-- (un)weighted\_unifrac\_tTest.{pdf,png} | 【组间 T-test 检验可视化展示（组内样本>2,组=2）】 |
| \| \| \| \| \|-- (un)weighted\_unifrac\_twoWilcox\_all.txt | 【组间 twowilcox 秩和检验（组内样本>2,组=2）】 |
| \| \| \| \| \|-- (un)weighted\_unifrac\_twoWilcox.{pdf,png} | 【组间 twowilcox 秩和检验可视化展示（组内样本>2,组=2）】 |
| \|-- 06.StatisticalTest | 【统计检验结果】 |
| \| \|-- adonis | 【 Adonis 分析结果】 |
| \| \| \|-- group1/... | 【分组 Adonis 分析结果】 |
| \| \| \| \|-- bray\_adonis.txt | 【 Adonis 分析结果】 |
| \| \|-- anosim | 【 Anosim 分析结果】 |
| \| \| \|-- group1/... | 【分组 Anosim 分析结果】 |
| \| \| \| \|-- stat\_anosim.txt | 【 Anosim 分析结果】 |
| \| \|-- mrpp | 【 MRPP 分析结果】 |
| \| \| \|-- group1/... | 【分组 MRPP 分析结果】 |
| \| \| \| \|-- stat\_mrpp.txt | 【 MRPP 分析结果】 |
| \| \|-- metagenomeseq | 【 Metagenomeseq 分析结果】 |
| \| \| \|-- group1/... | 【分组下Metagenomeseq 分析结果】 |
| \| \| \| \|-- phylum/... | 【门纲目科属种（p,c,o,f,g,s）水平上的Metagenomeseq 分析结果】 |
| \| \| \| \| \|-- diffHeatmap | 【 top10 丰度物种的 heatmap 热图分析结果】 |
| \| \| \| \| \| \|-- annotation.heatmap.{pdf,png} | 【具有显著性差异物种的 top10 丰度的 heatmap 热图分析结果】 |
| \| \| \| \| \|-- \*-\* | 【组间 Metagenomeseq 分析结果】 |
| \| \| \| \| \| \|-- \*.psig.xls | 【从 Metagenomeseq 分析结果中，筛选出的 pvalues<=0.05 的信息】 |
| \| \| \| \| \| \|-- \*.qsig.xls | 【从 \*.psig.xls 分析结果中，筛选出的 adjPvalues<=0.05 的信息】 |
| \| \| \| \| \| \|-- \*.Volcano.{pdf,png} | 【具有显著性差异物种未标样品名称的火山图】 |
| \| \| \| \| \| \|-- \*.Volcano..label.{pdf,png} | 【具有显著性差异物种标有样品名称的火山图】 |
| \| \| \| \| \| \|-- boxplot | 【具有显著性差异物种的箱图结果】 |
| \| \| \| \| \| \| \|-- \*.{pdf,png} | 【显著性差异物种的箱图结果展示】 |
| \| \|-- simper | 【物种对样本差异贡献度分析（simper分析）】 |
| \| \| \|-- group1/... | 【分组下物种对样本差异贡献度分析（simper分析）】 |
| \| \| \| \|-- Rplots.pdf | 【种（s）水平上物种对样本差异贡献度分析合并图】 |
| \| \| \| \|-- phylum/... | 【门纲目科属种（p,c,o,f,g,s）水平上物种对样本差异贡献度分析】 |
| \| \| \| \| \|-- \*-\* | 【组间 top10 物种对样本差异贡献度分析】 |
| \| \| \| \| \| \|-- \*-\*.{xls,pdf,png} | 【组间 top10 物种对样本差异贡献度分析结果及可视化】 |
| \| \|-- ttest | 【组间差异显著的物种分析】 |
| \| \| \|-- group1/... | 【组间差异显著的物种分析】 |
| \| \| \| \|-- phylum/... | 【门纲目科属种（p,c,o,f,g,s）水平上组间差异显著的物种分析】 |
| \| \| \| \| \|-- \*-\* | 【组间差异显著的物种分析】 |
| \| \| \| \| \| \|-- \*.psig.xls | 【两组之间的t-test数据结果，筛选出的 p value<=0.05 的信息】 |
| \| \| \| \| \| \|-- \*.qsig.xls | 【两组之间的t-test数据结果，筛选出的 q value<=0.05 的信息】 |
| \| \| \| \| \| \|-- \*.Volcano.{pdf,png} | 【具有显著性差异物种未标样品名称的火山图】 |
| \| \| \| \| \| \|-- \*.Volcano..label.{pdf,png} | 【具有显著性差异物种标有样品名称的火山图】 |
| \| \| \| \| \| \|-- boxplot | 【具有显著性差异物种的箱图结果】 |
| \| \| \| \| \| \| \|-- \*.{pdf,png} | 【显著性差异物种的箱图结果展示】 |
| \| \|-- lefse | 【LEfSe 分析结果】 |
| \| \| \|-- group1/... | 【组间 LEfSe 分析结果】 |
| \| \| \| \|-- \*-\* | 【组间 LEfSe 分析结果】 |
| \| \| \| \| \|-- LDA.\*.all.res | 【 LEfSe 统计结果】 |
| \| \| \| \| \|-- LDA.\*.draw.res | 【 LEfSe 作图数据】 |
| \| \| \| \| \|-- LDA.\*.{svg,png} | 【 LDA 值分布柱状图】 |
| \| \| \| \| \|-- LDA.\*.tree.{svg,png} | 【 LEfSe 进化分支图】 |
| \| \|-- KruskalWallis | 【KruskalWallis 组间差异检验结果】 |
| \| \| \|-- group1/... | 【KruskalWallis 组间差异检验结果】 |
| \| \| \| \|-- phylum/... | 【门纲目科属种（p,c,o,f,g,s）水平上KruskalWallis组间差异检验】 |
| \| \| \| \| \|-- \*-\* | 【指定组间 KruskalWallis 差异检验结果】 |
| \| \| \| \| \| \|-- KruskalWallis\_all.xls | 【组间所有差异显著的物种结果】 |
| \| \| \| \| \| \|-- KruskalWallis\_sig.xls | 【组间p value<0.05的差异显著的物种结果】 |
| \| \| \| \| \| \|-- KruskalWallis\_dunn.xls | 【Dunn多重检验的结果】 |
| \| \| \| \| \| \|-- KruskalWallis\_top\*.xls | 【各层级下平均相对丰度排名前10的物种差异检验结果】 |
| \| \| \| \| \| \|-- KruskalWallis\_top\_releative.xls | 【各层级下平均相对丰度排名前10的物种丰度表】 |
| \| \| \| \| \| \|-- kw\_barplot.{pdf,png} | 【KruskalWallis检验的平均丰度柱状图结果】 |
| \| \| \| \| \| \|-- kw\_boxplot.{pdf,png} | 【KruskalWallis检验的平均丰度箱形图结果】 |
| \| \|-- Wilcoxon | 【Wilcoxon 组间差异检验结果】 |
| \| \| \|-- group1/... | 【Wilcoxon 组间差异检验结果】 |
| \| \| \| \|-- phylum/... | 【门纲目科属种（p,c,o,f,g,s）水平上Wilcoxon组间差异检验】 |
| \| \| \| \| \|-- \*-\* | 【指定组间 Wilcoxon 差异检验结果】 |
| \| \| \| \| \| \|-- Wilcoxon\_all.xls | 【组间所有差异显著的物种结果】 |
| \| \| \| \| \| \|-- Wilcoxon\_sig.xls | 【组间p value<0.05的差异显著的物种结果】 |
| \| \| \| \| \| \|-- Wilcoxon\_top\*.xls | 【各层级下平均相对丰度排名前10的物种差异检验结果】 |
| \| \| \| \| \| \|-- Wilcoxon\_top\_releative.xls | 【各层级下平均相对丰度排名前10的物种丰度表】 |
| \| \| \| \| \| \|-- wilcoxon\_barplot.{pdf,png} | 【Wilcoxon检验的平均丰度柱状图结果】 |
| \| \| \| \| \| \|-- wilcoxon\_boxplot.{pdf,png} | 【Wilcoxon检验的平均丰度箱形图结果】 |
| \| \|-- randomForest | 【随机森林结果】 |
| \| \| \|-- group1/... | 【组间随机森林结果】 |
| \| \| \| \|-- class/... | 【纲目科属种（c,o,f,g,s）水平上随机森林结果】 |
| \| \| \| \| \|-- trainset\_group\_max\_roc.{pdf,png} | 【各变量训练集最大AUC值,绘制ROC曲线组合图】 |
| \| \| \| \| \|-- \*-\* | 【组间随机森林结果】 |
| \| \| \| \| \| \|-- imptop10MD.{pdf,png} | 【 top10 变量平均精度下降图形展示】 |
| \| \| \| \| \| \|-- imptop10MG.{pdf,png} | 【 top10 变量平均 Gini 指数下降图形展示】 |
| \| \| \| \| \| \|-- testset\_auc.{pdf,png} | 【各重要变量测试集 ROC 曲线组合图形展示】 |
| \| \| \| \| \| \|-- testset.point\_auc.{pdf,png} | 【各重要变量测试集 AUC 点图图形展示】 |
| \| \| \| \| \| \|-- trainset\_auc.{pdf,png} | 【各重要变量训练集 ROC 曲线组合图形展示】 |
| \| \| \| \| \| \|-- trainset.point\_auc.{pdf,png} | 【各重要变量训练集 AUC 点图图形展示】 |
| \| \| \| \| \| \|-- 3/5/... | 【选取 num（3，5，10，50，70，100，150，200）个最重要变量的数目构建随机森林模型】 |
| \| \| \| \| \| \| \|-- cross\_validation\_pick\_\*.im.var.txt | 【通过交叉验证得到的重要变量特征】 |
| \| \| \| \| \| \| \|-- cverrof.{pdf,png} | 【变量筛选数目与错误率图形展示】 |
| \| \| \| \| \| \| \|-- impplot\_MeanDecreaseAccuracy\*.{pdf,png} | 【平均精度下降图形展示】 |
| \| \| \| \| \| \| \|-- impplot\_MeanDecreaseGin\*.{pdf,png} | 【平均 Gini 指数下降图形展示】 |
| \| \| \| \| \| \| \|-- testset.probabilty.xls | 【随机森林模型对测试集样本的预测概率】 |
| \| \| \| \| \| \| \|-- testset.ROC.{pdf,png} | 【测试集 ROC 曲线图形展示】 |
| \| \| \| \| \| \| \|-- testset.roc.xls | 【测试集 AUC 值 1 代表平均值 2 代表最小最 3 代表最大值】 |
| \| \| \| \| \| \| \|-- top\_\*testset.var\_predict\_box\_plot.{pdf,png} | 【构建随机森林模型时,测试集预测的准确率图形展示】 |
| \| \| \| \| \| \| \|-- top\_\*trainset.var\_predict\_box\_plot.{pdf,png} | 【构建随机森林模型时,训练集预测的准确率图形展示】 |
| \| \| \| \| \| \| \|-- top\_\*var\_imp.xls | 【选取的n个重要变量分别对样本的贡献度及对模型构建的影响程度】 |
| \| \| \| \| \| \| \|-- trainset.probabilty.xls | 【随机森林模型对训练集样本的预测概率】 |
| \| \| \| \| \| \| \|-- trainset.ROC.{pdf,png} | 【训练集 ROC 曲线图形展示】 |
| \| \| \| \| \| \| \|-- trainset.roc.xls | 【训练集 AUC 值 1 代表平均值 2 代表最小最 3 代表最大值】 |
| \|-- 07.CorrelationAnalysis | 【关联分析】 |
| \| \|-- network | 【 network 分析结果】 |
| \| \| \|-- group1/... | 【组间 network 分析结果】 |
| \| \| \| \|-- \*/dot/genus.{png,svg} | 【network结果图形展示】 |
| \| \| \| \|-- \*/dot/genus.spearman.index.list | 【network 网页图绘制边文件】 |
| \| \| \| \|-- \*/dot/node.xls | 【network 网页图绘制节点文件】 |
| \| \| \| \|-- \*/dot/igraph.calculate.txt | 【network图中的关键参数展示，如网络图直径（ND），平均连接度（AD）等】 |
| \| \| \|-- sample/... | 【样本间 network 分析结果】 |
| \| \| \| \|-- dot/genus.{png,svg} | 【network结果图形展示】 |
| \| \| \| \|-- dot/genus.spearman.index.list | 【network 网页图绘制边文件】 |
| \| \| \| \|-- dot/node.xls | 【network 网页图绘制节点文件】 |
| \| \| \| \|-- dot/igraph.calculate.txt | 【network图中的关键参数展示，如网络图直径（ND），平均连接度（AD）等】 |
| \| \|-- network3D | 【 network3D 分析结果】 |
| \| \| \|-- group1/... | 【组间 network 分析结果】 |
| \| \| \| \|-- \*/cytoplot.csv | 【network3D 本地化Cytoscape绘制网络图数据】 |
| \| \| \| \|-- \*/edge.xls | 【network3D 网页图绘制边文件】 |
| \| \| \| \|-- \*/node.xls | 【network3D 网页图绘制节点文件】 |
| \| \| \| \|-- netfig | 【 network3D 网页展示文件】 |
| \| \| \|-- sample/... | 【样本间 network 分析结果】 |
| \| \| \| \|-- cytoplot.csv | 【network3D 本地化Cytoscape绘制网络图数据】 |
| \| \| \| \|-- edge.xls | 【network3D 网页图绘制边文件】 |
| \| \| \| \|-- node.xls | 【network3D 网页图绘制节点文件】 |
| \| \| \| \|-- netfig | 【 network3D 网页展示文件】 |
| \|-- 08.FunctionPrediction | 【功能预测结果】 |
| \| \|-- picrust2 | 【 picrust2 预测结果】 |
| \| \| \|-- predicted | 【 picrust2（COG,EC,KO,PFAM,TIGRFAM,pathway）数据库预测结果】 |
| \| \| \| \|-- \*\_metagenome\_descrip.tsv | 【（COG,EC,KO,PFAM,TIGRFAM,pathway）数据库预测结果】 |
| \| \| \| \|-- \*\_seqtab\_norm.tsv.gz | 【基于预测 16S 拷贝数校正的特征表, pathways\_out 无此结果】 |
| \| \| \| \|-- \*\_weighted\_nsti.tsv.gz | 【每个样本的 NSTI 权重, pathways\_out 无此结果】 |
| \| \| \|-- tables | 【预测结果的相对（绝对）丰度】 |
| \| \| \| \|-- sample/group1/... | 【样本及分组 预测结果的相对（绝对）丰度】 |
| \| \| \| \| \|-- picrust2.{sample,group}.\*.absolute.xls | 【(样本,分组)各数据库预测结果的绝对丰度】 |
| \| \| \| \| \|-- picrust2.{sample,group}.\*.relative.xls | 【(样本,分组)各数据库预测结果的相对丰度】 |
| \| \| \|-- barplot | 【功能相对丰度柱形图，展示同物种barplot】 |
| \| \| \|-- heatmap | 【功能注释聚类热图，展示同物种heatmap】 |
| \| \| \|-- venn | 【基于功能的venn结果，展示同物种venn】 |
| \| \| \|-- flower | 【基于功能的花瓣图，展示同物种flower】 |
| \| \| \|-- pca | 【各功能 PCA 分析结果，展示同beta\_pca】 |
| \| \| \|-- ttest | 【组间差异显著的功能分析各，展示同物种ttest】 |
| \| \|-- picrust | 【 picrust 预测结果】 |
| \| \| \|-- predicted | 【 KEGG 预测结果】 |
| \| \| \| \|-- KEGG\_metagenome\_predictions.{biom,txt} | 【 KEGG 预测结果】 |
| \| \| \| \|-- predicted\_metagenomes.KEGG\_L\*.txt | 【 KEGG（1,2,3）层级预测结果】 |
| \| \| \|-- tables | 【预测结果的相对（绝对）丰度】 |
| \| \| \| \|-- sample/group1/... | 【样本及分组 预测结果的相对（绝对）丰度】 |
| \| \| \| \| \|-- picrust.{sample,group}.\*.absolute.xls | 【(样本,分组)（1,2,3,k）层级预测结果的绝对丰度】 |
| \| \| \| \| \|-- picrust.{sample,group}.\*.relative.xls | 【(样本,分组)（1,2,3,k）层级预测结果的相对丰度】 |
| \| \| \|-- barplot | 【功能相对丰度柱形图，展示同物种barplot】 |
| \| \| \|-- heatmap | 【功能注释聚类热图，展示同物种heatmap】 |
| \| \| \|-- venn | 【基于功能的venn结果，展示同物种venn】 |
| \| \| \|-- flower | 【基于功能的花瓣图，展示同物种flower】 |
| \| \| \|-- pca | 【各功能 PCA 分析结果，展示同beta\_pca】 |
| \| \| \|-- ttest | 【组间差异显著的功能分析各，展示同物种ttest】 |
| \| \|-- tax4fun | 【 tax4fun 预测结果】 |
| \| \| \|-- predicted | 【 KEGG 预测结果】 |
| \| \| \| \|-- KEGG\_metagenome\_predictions.txt | 【 KEGG 预测结果】 |
| \| \| \| \|-- predicted\_metagenomes.KEGG\_L\*.txt | 【 KEGG（1,2,3）层级预测结果】 |
| \| \| \|-- tables | 【预测结果的相对（绝对）丰度】 |
| \| \| \| \|-- sample/group1/... | 【样本及分组 预测结果的相对（绝对）丰度】 |
| \| \| \| \| \|-- tax4fun.{sample,group}.\*.absolute.xls | 【(样本,分组)（1,2,3,k）层级预测结果的绝对丰度】 |
| \| \| \| \| \|-- tax4fun.{sample,group}.\*.relative.xls | 【(样本,分组)（1,2,3,k）层级预测结果的相对丰度】 |
| \| \| \|-- barplot | 【功能相对丰度柱形图，展示同物种barplot】 |
| \| \| \|-- heatmap | 【功能注释聚类热图，展示同物种heatmap】 |
| \| \| \|-- venn | 【基于功能的venn结果，展示同物种venn】 |
| \| \| \|-- flower | 【基于功能的花瓣图，展示同物种flower】 |
| \| \| \|-- pca | 【各功能 PCA 分析结果，展示同beta\_pca】 |
| \| \| \|-- ttest | 【组间差异显著的功能分析各，展示同物种ttest】 |
| \| \|-- bugbase | 【 bugbase 预测结果】 |
| \| \| \| \|-- normalized\_otus | 【标准化 OTU 表】 |
| \| \| \| \|-- otu\_contributions | 【表型预测结果堆叠柱形图】 |
| \| \| \| \| \|-- \*.{pdf,png} | 【表型预测结果堆叠柱形图】 |
| \| \| \| \| \|-- contributing\_otus.txt | 【表型预测结果表】 |
| \| \| \| \| \|-- otu\_table.relative.xls | 【表型预测结果相对丰度表】 |
| \| \| \| \|-- predicted\_phenotypes | 【表型预测结果比较箱线图】 |
| \| \| \| \| \|-- \*.{pdf,png} | 【表型预测结果比较箱线图】 |
| \| \| \| \| \|-- \*\_stats.txt | 【表型预测结果箱型图绘制参数，如平均数，中位数，标准差】 |
| \| \| \| \| \|-- predictions.{txt,xls} | 【样本表型丰度表】 |
| \| \| \| \|-- thresholds | 【阈值选择表】 |
| \| \| \| \| \|-- variances.txt | 【不同阈值下的结果】 |
| \| \| \| \| \|-- thresholds\_used.txt | 【表型预测分析使用的阈值】 |
| \| \| \| \| \|-- \*.{pdf,png} | 【9表型在不同阈值下相对丰度变化】 |
| \| \|-- funguild | 【 funguild 预测结果】 |
| \| \| \|-- predicted | 【 funguild 预测结果】 |
| \| \| \| \|-- funguild.sample.total.absolute.xls | 【 funguild 预测结果】 |
| \| \| \| \|-- funguild.sample.total.absolute.{guild, mode} | 【 funguild（guild,mode）预测结果】 |
| \| \| \|-- tables | 【预测结果的相对（绝对）丰度】 |
| \| \| \| \|-- sample/group1/... | 【样本及分组 预测结果的相对（绝对）丰度】 |
| \| \| \| \| \|-- funguild.{sample,group}.\*.absolute.xls | 【(样本,分组)（total,guild,mode）预测结果的绝对丰度】 |
| \| \| \| \| \|-- funguild.{sample,group}.\*.relative.xls | 【(样本,分组)（total,guild,mode）预测结果的相对丰度】 |
| \| \| \|-- barplot | 【功能相对丰度柱形图，展示同物种barplot】 |
| \| \| \|-- heatmap | 【功能注释聚类热图，展示同物种heatmap】 |
| \| \| \|-- venn | 【基于功能的venn结果，展示同物种venn】 |
| \| \| \|-- flower | 【基于功能的花瓣图，展示同物种flower】 |
| \| \| \|-- pca | 【各功能 PCA 分析结果，展示同beta\_pca】 |
| \| \| \|-- ttest | 【组间差异显著的功能分析各，展示同物种ttest】 |
| \| \|-- faprotax | 【 faprotax 预测结果】 |
| \| \| \|-- predicted | 【 funguild 预测结果】 |
| \| \| \| \|-- faprotax.report.txt | 【 faprotax 预测结果】 |
| \| \| \| \|-- faprotax.sample.anno.absolute.xls | 【 faprotax 预测结果绝对丰度】 |
| \| \| \|-- tables | 【预测结果的相对（绝对）丰度】 |
| \| \| \| \|-- sample/group1/... | 【样本及分组 预测结果的相对（绝对）丰度】 |
| \| \| \| \| \|-- faprotax.{sample,group}.anno.absolute.xls | 【(样本,分组)预测结果的绝对丰度】 |
| \| \| \| \| \|-- faprotax.{sample,group}.anno.relative.xls | 【(样本,分组)预测结果的相对丰度】 |
| \| \| \|-- barplot | 【功能相对丰度柱形图，展示同物种barplot】 |
| \| \| \|-- heatmap | 【功能注释聚类热图，展示同物种heatmap】 |
| \| \| \|-- venn | 【基于功能的venn结果，展示同物种venn】 |
| \| \| \|-- flower | 【基于功能的花瓣图，展示同物种flower】 |
| \| \| \|-- pca | 【各功能 PCA 分析结果，展示同beta\_pca】 |
| \| \| \|-- ttest | 【组间差异显著的功能分析各，展示同物种ttest】 |
| \|-- 09.EnvironmentFactor | 【环境因子分析】 |
| \| \|--CCA | 【CCA 分析结果】 |
| \| \| \|--CCA\_standard | 【包含所有环境因子的CCA和RDA】 |
| \| \| \|--CCA\_FitENV | 【经envfit筛选后环境因子的CCA和RDA】 |
| \| \| \|--CCA\_VIF | 【经VIF筛选后环境因子的CCA和RDA】 |
| \| \| \|--CCA\_BioENV | 【经BioENV筛选后环境因子的CCA和RDA】 |
| \| \| \|--BioENV.txt | 【经Bioenv筛选后得到的环境因子组合】 |
| \| \| \|--VIF.txt | 【经VIF筛选后得到的环境因子组合】 |
| \| \| \|--selected\_FitENV.list | 【经FitENV筛选后得到的环境因子组合】 |
| \| \|--mantelTest | 【门纲目科属种以及otu（p,c,o,f,g,s,otu）水平上的mantel\_test 分析结果】 |
| \| \|--spearman | 【门纲目科属种和otu以及alpha（p,c,o,f,g,s,otu,alpha）水平上的 spearman 分析结果,属水平目录下含有环境因子与丰度的玫瑰图展示】 |
| \| \|--vpa | 【VPA 分析结果】 |
| \| \| \|--VPA1.{png,svg} | 【VPA分组环境因子解释率图】 |
| \|-- 10.CommAssembly | 【群落组装分析】 |
| \| \|-- bNTI | 【βNTI 分析】 |
| \| \| \|-- group1/... | 【分组 βNTI 分析结果】 |
| \| \| \| \|-- bNTI.summary.xls | 【βNTI 分组计算结果的统计数据】 |
| \| \| \| \|-- bNTI.xls | 【βNTI 所有样本对计算结果】 |
| \| \| \| \|-- bNTI.boxplot.{pdf,png} | 【βNTI 分析结果箱型图可视化】 |
| \| \|-- nst | 【NST 分析】 |
| \| \| \|-- group1/... | 【分组 taxonomic NST 分析结果】 |
| \| \| \| \|-- tNST.summary.xls | 【tNST 分析结果统计数据】 |
| \| \| \| \|-- tNST.pairwise.xls | 【tNST 所有样本对计算结果】 |
| \| \| \| \|-- tNST.boxplot.{pdf,png} | 【tNST 分析结果箱型图可视化】 |
| \| \| \| \|-- tNST.PERMANOVA.xls | 【tNST 分析PERMANOVA检验】 |
| \| \| \| \|-- tNST.boot.summary.xls | 【tNST 的 bootstrapping 自助重采样结果统计数据】 |
| \| \| \| \|-- tNST.boot.compare.xls | 【tNST 的 bootstrapping 自助重采样结果统计数据】 |
| \| \| \| \|-- tNST.boots.{pdf,png} | 【tNST 的 bootstrapping 自助重采样结果小提琴散点图可视化】 |
| \| \| \| \|-- tNST.concatenate.{pdf,png} | 【tNST 箱型图和小提琴散点图的结合可视化】 |
| \| \| \| \|-- pNST.summary.xls | 【pNST 分析结果统计数据】 |
| \| \| \| \|-- pNST.pairwise.xls | 【pNST 所有样本对计算结果】 |
| \| \| \| \|-- pNST.boxplot.{pdf,png} | 【pNST 分析结果箱型图可视化】 |
| \| \| \| \|-- pNST.PERMANOVA.xls | 【pNST 分析PERMANOVA检验】 |
| \| \| \| \|-- pNST.boot.summary.xls | 【pNST 的 bootstrapping 自助重采样结果统计数据】 |
| \| \| \| \|-- pNST.boot.compare.xls | 【pNST 的 bootstrapping 自助重采样结果统计数据】 |
| \| \| \| \|-- pNST.boots.{pdf,png} | 【pNST 的 bootstrapping 自助重采样结果小提琴散点图可视化】 |
| \| \| \| \|-- pNST.concatenate.{pdf,png} | 【pNST 箱型图和小提琴散点图的结合可视化】 |
| \| \|-- icamp | 【iCAMP 分析】 |
| \| \| \|-- group1/... | 【分组 iCAMP 分析结果】 |
| \| \| \| \|-- icamp.ProcessImportance\_EachGroup.csv | 【iCAMP 分析的各分组组装机制重要性结果】 |
| \| \| \| \|-- icamp.ProcessImportance\_EachTurnover.csv | 【iCAMP 分析的各分组所有样本对组装机制重要性结果】 |
| \| \| \| \|-- icamp.circle\_{group}\_Group.{pdf,png} | 【iCAMP 分析各组重要性数据圈图可视化】 |
| \| \| \| \|-- icamp.barEachGroup.{pdf,png} | 【iCAMP 分析所有分组重要性数据柱状图可视化】 |
|  |  |

`Tips:`

- Metagenomeseq、Adonis、MRPP、Anosim、t.test、LEfSe、KruskalWallis和Wilcoxon等组间差异分析，每个分组不少于三个生物学重复时才能做相应的统计检验（若组间无差异物种，则相应的检验结果不展示）。
- 群落组装分析要求存在分组且不少于三个生物学重复，否则无此分析。
- 随机森林要求每个分组不少于15个生物学重复，否则无此分析。
- 功能预测和环境因子部分需在搜集表中填写，若未填写相关分析则对应结果不展示。
- network分析，默认样本数不少于六个时方可出图。

  ​

---

```
```
xxxxxxxxxx
```


```
                           如有疑问请咨询项目销售或运营，我们将竭诚为您服务。
```
```
